# 

**Published:** 1923-03

**Authors:** 


### Contents

Event and Comment ........ 97
A Few Remarks on the Role of the American Dentists in Paris During the Great War ........ 99
Cuspid Relationship and Pyorrhea Alveolaris ........ 112
What the American Medical Association Thinks of the Electronic Reaction of Abrams ........ 117
History of a Case of Carcinoma of the Antrum ........ 125
A Message from Thwaites ........ 126
Defective Teeth and Nutrition ........ 127
The S. S. White Electric Furnace "B" with Pyrometer ........ 131
Indents ........ 134
Announcements ........ 138

